# Differences in the expression of long noncoding RNAs at different time points in the PTSD-like syndrome rat hippocampus

**DOI:** 10.18632/oncotarget.21025

**Published:** 2017-09-18

**Authors:** Qingzhen Liu, Qing Ji, Jian Liu, Lidong Zhang

**Affiliations:** ^1^ Department of Anesthesiology, Jinling Hospital, School of Medicine, Nanjing University, Nanjing, 210002, China

**Keywords:** long non-coding RNAs(lncRNAs), PTSD, EU056364_P1, Camk2a, expression signature

## Abstract

The aim of this study was to characterize the expression profiles at different time points in the PTSD-like syndrome rat hippocampus and perform analyses. PTSD rat models were made as reported by Rau, and we collected the hippocampus at different time points. The lncRNAs at different time points were compared by microarray and listed. We used quantitative real-time PCR to confirm the lncRNA profiling expression data. Bioinformatics analysis was performed on EU056364_P1. Compare with control, a total of 948 lncRNAs and 2514 mRNAs were found (fold-change > 2.0) among the four time points. Additionally, bioinformatics analysis of EU056364_P1 suggested it might be involved in memory development through the target gene Camk2a.This study revealed different lncRNAs expressed at different time points in PTSD and explored the targets of PTSD.

## INTRODUCTION

Posttraumatic stress disorder (PTSD) is characterized by hypermnesia of the trauma with memory impairment. Avoidance, increased alertness, emotional numbing and re-experiencing of the trauma are its core symptoms. The rate of PTSD in individuals after experiencing threatening events such as intraoperative awareness is approximately 2–9% [1–2]. PTSD affects patients’ social and family life. We need to find effective treatments for PTSD, and it is a hotspot of medical research. There are currently no specific drugs for PTSD[3]. This study aimed to identify differences in lncRNA expression among the four groups and reveal a new mechanism to cure PTSD.

Long non-coding RNAs(LncRNAs) are a type of non-coding RNA that can regulate gene expression in many diseases, including schizophrenia and major depression [4]. Many of them have been confirmed to perform regulatory functions relevant to these disease processes [5]. However, the detailed mechanisms of PTSD in animal models remain unclear. Now, we explore the connection between lncRNAs and PTSD-like syndrome.

## RESULTS

### Behaviour test

**Figure 1:**
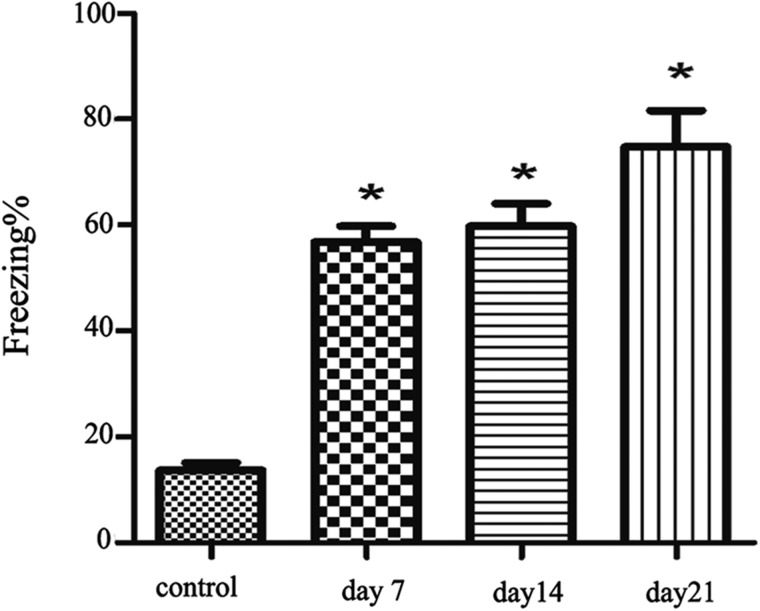
The freezing of the four group

Compared with the control group, the samples frozen on day 7, day 14, and day 21 show significant Freezing enhancement (*P* < 0.05; Figure 1), which shows that the PTSD model is successful.

### LncRNA microarray profiling

**Figure 2:**
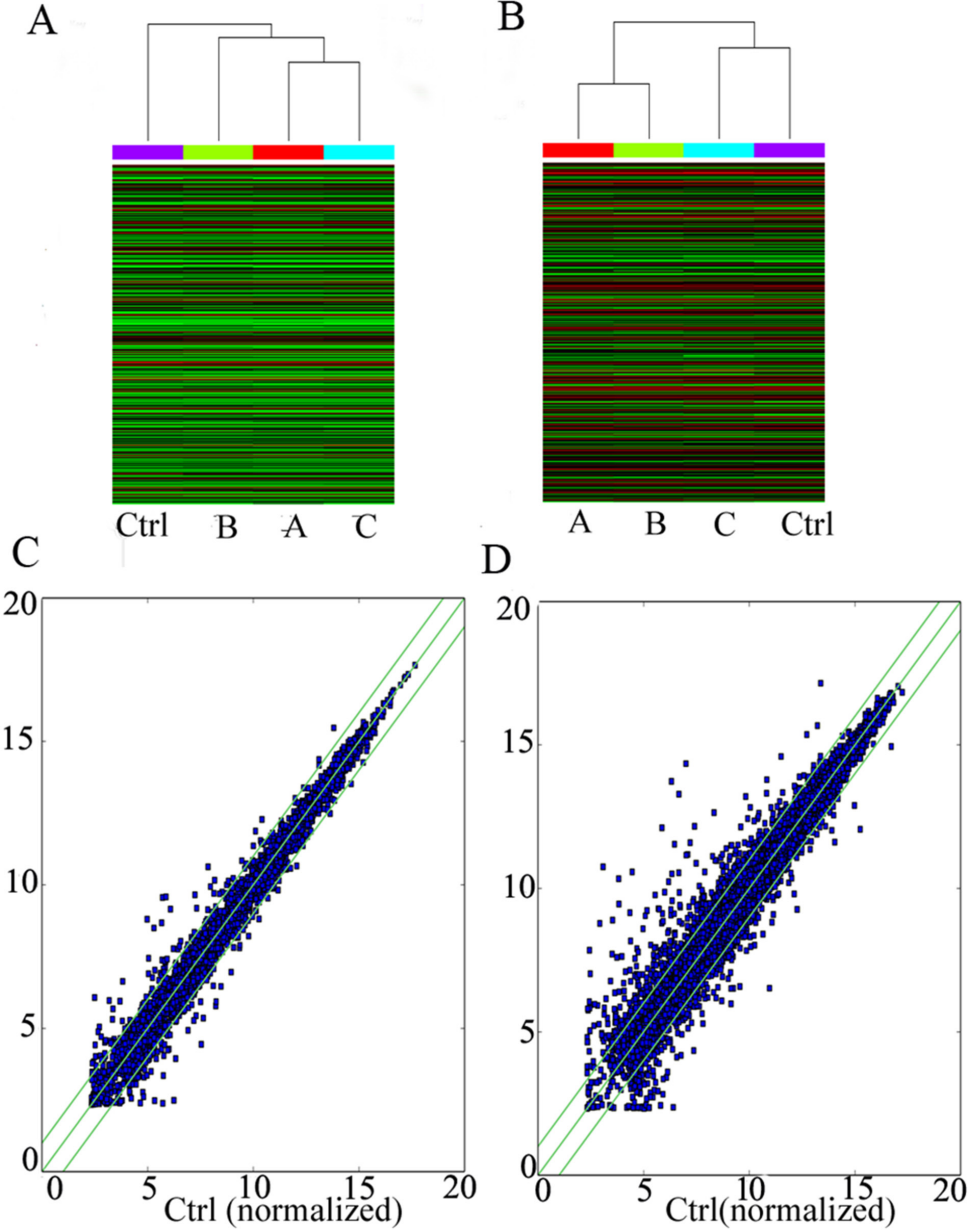
LncRNA microarray profiling data The lncRNA and mRNA microarray expression data for the four PTSD-like syndrome groups. (**A**) Hierarchical clustering shows distinguishable lncRNA expression profiles among groups. (**B**) Hierarchical clustering shows distinguishable mRNA expression profiles among groups.(**C**) The points above the top green line and below the bottom green line indicate more than a 2.0-fold change in the lncRNAs among the four PTSD-like syndrome groups. (**D**) The mRNAs showing more than 2.0-fold change are also shown above the top green line and below the bottom green line.

Using a second-generation lncRNA microarray, 948 lncRNAs and 2514 coding transcripts were detected (Table 1). These transcripts were carefully identified using the most authoritative databases, such as UCSC. The scatter-plot visualization method was used to assess the lncRNA and mRNA expression variation among the four groups of PTSD-like syndrome rat (Figure 2C and 2D). Hierarchical clustering shows the lncRNA and mRNA expression patterns of the samples (Figure 2A and 2B).

**Table 1:** Expression of lncRNAs and mRNAs in different groups (> = 2.0-fold change)

group	Long noncoding RNA		mRNA	
	up	down	up	down
day7 vs Ctrl	101	109	495	317
day 14 vs Ctrl	150	295	464	562
day21 vs Ctrl	143	150	267	409
Total	394	554	1226	1288

### Pathway analysis

**Figure 3:**
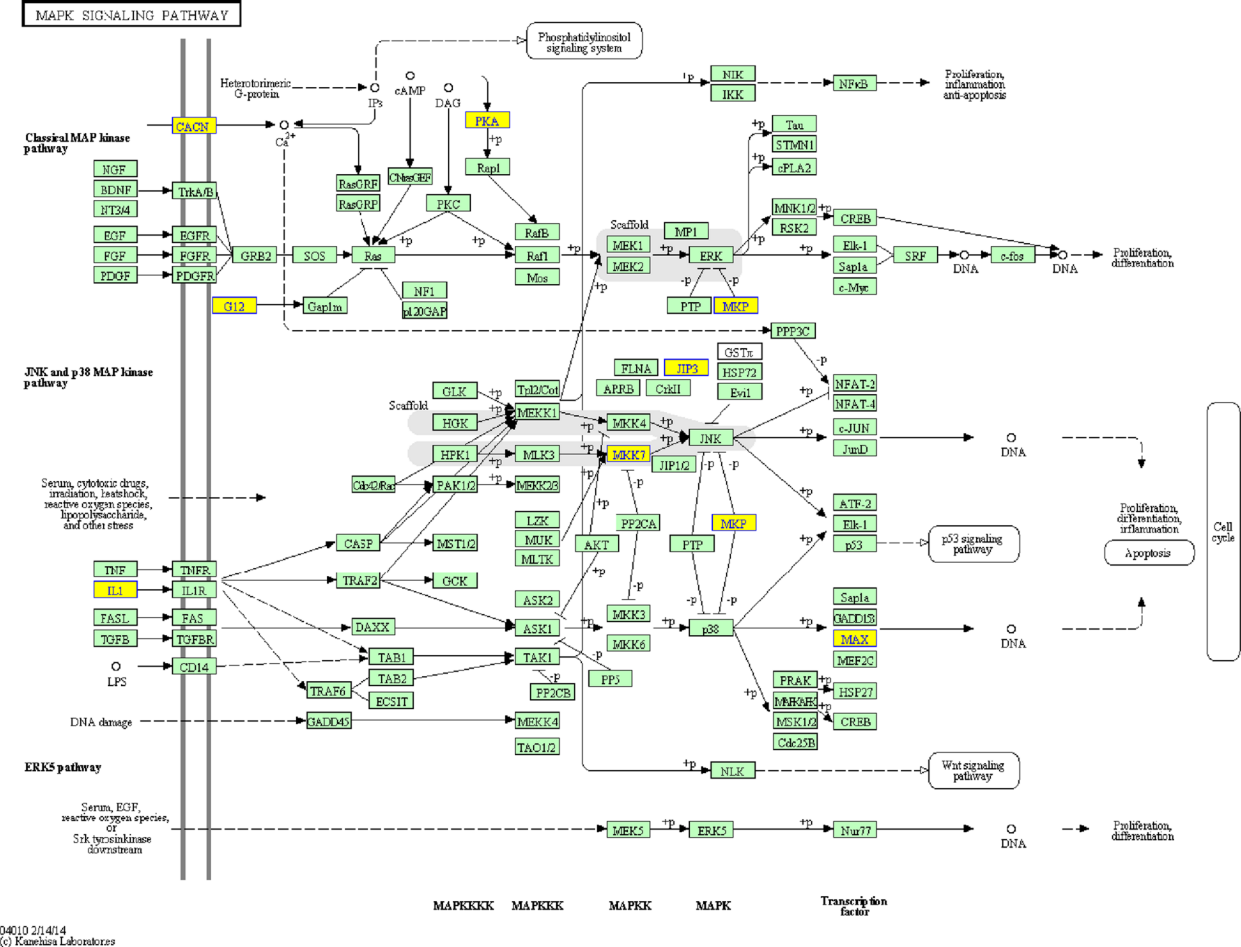
Example pathway: the MAPK signalling pathway

We performed pathway analysis by mapping genes to KEGG pathways (http://www.genome.jp/kegg/). In our study, we show 10 pathways that include coding genes associated with the lncRNAs involved in PTSD (Figure 3). The recommended *P*-value cut-off is 0.05.

### GO analysis

**Figure 4:**
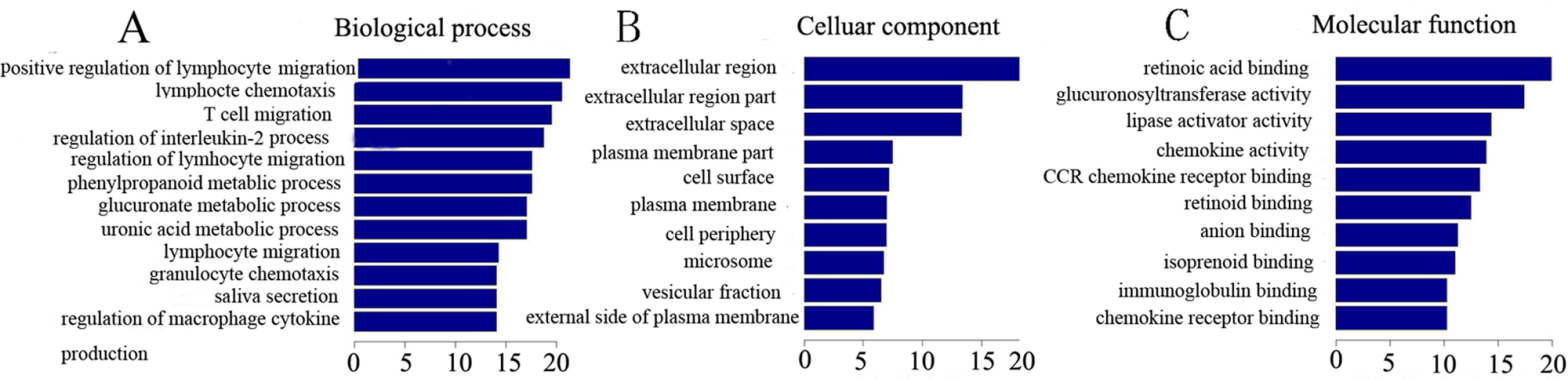
Gene enrichment of lncRNAs and coexpressed mRNAs The GO enrichment analysis provides a controlled vocabulary to describe the differentially expressed lncRNAs and coexpressed mRNAs. The ontology covers three domains: biological processes, cellular components, and molecular function. *P* < 0.05 is recommended.

The Gene Ontology project (http://www.geneontology.org) includes three domains: Molecular Functions, Biological Processes and Cellular Components. We present GO terms associated with the coding genes found in our below (Figure 4).

### Real-time quantitative PCR

**Figure 5:**
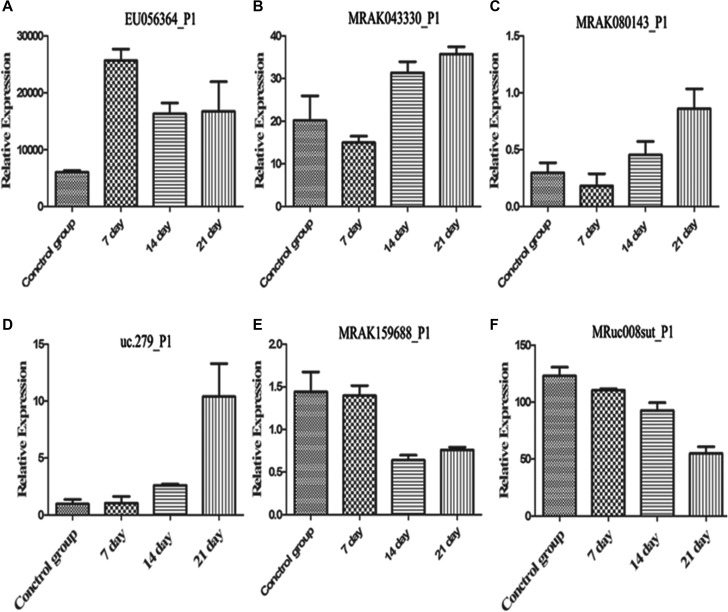
Quantitative real-time PCR was performed using hippocampus tissue samples ^*^p < 0.05.

We selected 6 differentially expressed lncRNAs (EU056364, DQ473607, uc.279-, MRAK080143, MRuc008sut, MRAK159688) from the different samples for each group (Figure 5) to confirm the microarray profiling expression data. The results indicate that similar up-regulation or down-regulation was observed in both the microarray and RT-PCR samples for all 6 lncRNAs. Therefore, our microarray data were reliable and stable.

## DISCUSSION

Our study reveals that many lncRNAs and mRNAs were significantly altered among the four PTSD-like syndrome groups. PTSD is a disorder that can occur upon exposure to an accident [7–10]. We used a second-generation lncRNA microarray to detect 394 up-regulated and 554 down-regulated lncRNAs in three PTSD-like syndrome groups compared to the control group (fold-change > 2.0). Different profiles of lncRNA and coding transcript expression were observed in PTSD-like syndrome rats at four time points and confirmed by RT-PCR, thus suggesting that numerous lncRNAs were involved in the development of PTSD-like syndrome conditions in rats.

Based on GO and KEGG Pathway analysis, many lncRNAs are closely related to the development of PTSD-like syndrome. The MAPK signalling pathways play important roles in PTSD, such as regulating synaptic plasticity and memory in the brain. There are three different families of MAPKs, namely ERK, p38-MAPK, and JNK-MAPK [11], with roles in stress response and memory [12, 13]. In mammals, the ERK/MAPK pathways are associated with memory tasks and participate in strengthening memory in the hippocampus [14].

Our results revealed that the gene associated with the lncRNA EU056364_P1 is CamkIIa. CaMKII plays a role in regulating synaptic plasticity and stimulates protein synthesis in the hippocampal neurons. It can regulate the early phase of LTP (long-term potentiation) [15] and participates in the course of LTP and fear-memory formation that underlies memory consolidation [16, 17, 18]. CaMKII [19, 20] is important in memory formation and extinction [21, 22].

LncRNAs can exhibit different expression patterns [23, 24]. Our study revealed lncRNA expression patterns at different time points of a PTSD-like syndrome rat model. some lncRNAs were down-regulated in early development and then up-regulated in later development; other lncRNAs were down-regulated in early development and then down-regulated again in later development; and still other lncRNAs were consistently down-regulated or up-regulated throughout PTSD development. These differential expression patterns may indicate involvement in the development of the PTSD-like syndrome rat model. These differential expression patterns may indicate involvement in the development of the PTSD-like syndrome rat model.

In conclusion, our study provides an expression profile of lncRNAs in the development of PTSD, as well as a series of differentially expressed miRNAs at different time points. This study clarify that lncRNAs play an important role in PTSD development and provide a physiological basis for future study of PTSD.

## MATERIALS AND METHODS

### Ethics statement

The experiment satisfied the requirements of the National Institutes of Health Guide to the Care and Use of Laboratory Animals and the Ethics Committee of Jinling Hospital(Nanjing,Jiangsu,China;approval ID:SYXK-2012-0047).

### Animals

Forty male adult Sprague-Dawley rats weighing 250-300 g were obtained from the Department of Laboratory Animal Science, Nanjing General Hospital of Nanjing Military Command. They were kept under a 12 h light-dark cycle with water and food pellets available ad libitum. The PTSD model was made as reported by Rau [6], and the hippocampus was collected afterward.

### Animal model

This experiment we adopted PTSD models as Rau [6] reported, Rats were used in this experiment. They were divided randomly into two groups. On day 1, animals were removed to context A (50 cm × 45 cm × 50 cm) and receive 0 or 15 shocks (1-mA, 1-s shocks with a variable intershock interval of 240–480 s,)Then the rats were removed from the chamber and placed in their home cages. On day 22, rats were given a single shock (1 mA, 1 s) 192 s after placement in context B (50 cm × 45 cm × 50 cm), and freezing for the 192s pre-shock period was assessed to provide a baseline prior to shock in context B. Freezing was defined as the absence of all movement except for respiration.

On 23 day, rats were returned to context B for 512 s for a test of fear conditioned higher than on 22 day's that we can determine the rats appeared SEFL - PTSD.

### Total RNA preparation

The total RNA from each sample was quantified using a NanoDrop ND-1000, and the RNA integrity was assessed by agarose gel electrophoresis.

### LncRNA expression analysis

For microarray analysis, we used the Agilent Array platform version 5.7 to amplify and transcribe the total RNA from each sample into fluorescent cRNA. The labelled cRNAs were hybridized using the Rat LncRNA 4 × 44K Array v2.0. We scanned the arrays using the Agilent Scanner G2505C and used the GeneSpring GX v11.5.1 software package and Agilent Feature Extraction software (version 11.0.1.1) to analyse the acquired array images and perform data processing.

### Pathway analysis

Based on the latest KEGG (Kyoto Encyclopedia of Genes and Genomes, http://www.genome.jp/kegg) database, we performed pathway analysis for differentially expressed mRNAs. This analysis allows users to determine biological pathways with a significant enrichment of differentially expressed mRNAs. The *P*-value denotes the significance of the pathway. The smaller the *P*-value, the more significant is the pathway. The *P*-value cut-off is 0.05.

### GO analysis

GO analysis is a functional analysis associating differentially expressed mRNAs with GO categories. The GO categories are derived from Gene Ontology (http://www.geneontology.org), which comprises three structured networks of defined terms that describe gene product attributes. The *P*-value denotes the significance of GO term enrichment in the list of differentially expressed mRNAs. The smaller the *P*-value, the more significant is the GO term (*P*-value < = 0.05 recommended).

### Quantitative real-time PCR

The cDNA was transcribed from 1 μg total RNA. Real-time PCR was performed using the SYBR green method. The PCR conditions included a denaturation step (95°C for 10 min) followed by 40 cycles of amplification and quantification (95°C for 15 s, 60°C for 1 min). The relative gene expression levels were quantified based on the cycle threshold (Ct) values and normalized to the reference gene GAPDH. The gene expression levels were calculated using the 2^-ΔΔCt^ method.

### Statistical analysis

All data were analysed using the SPSS 17.0 package (SPSS, Chicago, IL). The *t*-test and one-way or two-way analysis of variance (ANOVA) were used to analyse the expression levels of lncRNAs. *P* < 0.05 was considered significant.

## References

[1] Kessler RC, Berglund P, Demler O, Jin R, Merikangas KR, Walters EE (2005). Lifetime prevalence and age-of-onset distributions of DSM-IV disorders in the National Comorbidity Survey Replication. Arch Gen Psychiatry.

[2] Maercker A, Forstmeier S, Wagner B, Glaesmer H, Brähler E (2008). Post-traumatic stress disorder in Germany. Results of a nation-wide epidemiological study. Nervenarzt.

[3] Institute of Medicine (IOM) (2008). Treatment of posttraumatic stress disorder: An assessment of the evidence.

[4] Esteller M (2011). Non-coding RNAs in human disease. Nature Reviews Genetics.

[5] Gupta RA, Shah N, Wang KC, Kim J, Horlings HM, Wong DJ, Tsai MC, Hung T, Argani P, Rinn JL, Wang Y, Brzoska P, Kong B (2010). Long non-coding RNA HOTAIR reprograms chromatin state to promote cancer metastasis. Nature.

[6] Rau V, DeCola JP, Fanselow MS (2005). Stress-induced enhancement of fear learning: an animal model of posttraumatic stress disorder. Neuroscience & Biobehavioral Reviews.

[7] Rau V, Oh I, Laster M, Eger EI, Fanselow MS (2009). Isoflurane suppresses stress-enhanced fear learning in a rodent model of posttraumatic stress disorder. Anesthesiology.

[8] Fanselow MS (1980). Conditional and unconditional components of post-shock freezing. The Pavlovian Journal of Biological Science.

[9] American Psychiatric Association (2000). Diagnostic and Statistical Manual—Text Revision (DSM-IV-TR).

[10] Mahan AL, Ressler KJ (2012). Fear conditioning, synaptic plasticity and the amygdala: implications for posttraumatic stress disorder. Trends in Neurosciences.

[11] Johnson GL, Lapadat R (2002). Mitogen-activated protein kinase pathways mediated by ERK, JNK, and p38 protein kinases. Science.

[12] Bolshakov VY, Carboni L, Cobb MH, Siegelbaum SA, Belardetti F (2000). Dual MAP kinase pathways mediate opposing forms of longterm plasticity at CA3-CA1 synapses. Nat Neurosci.

[13] Grewal SS, York RD, Stork PJ (1999). Extracellular-signal-regulated kinase signalling in neurons. Curr Opin Neurobiol.

[14] Atkins CM, Selcher JC, Petraitis JJ, Trzaskos JM, Sweatt JD (1998). The MAPK cascade is required for mammalian associative learning. Nat Neurosci.

[15] Bevilaqua LR, Medina JH, Izquierdo I, Cammarota M (2005). Memory consolidation induces N-methyl-D-aspartic acid-receptor- and Ca2+/calmodulin-dependent protein kinase II-dependent modifications in alpha-amino-3-hydroxy-5-methylisoxazole-4-propionic acid receptor properties. Neuroscience.

[16] Izquierdo I, Bevilaqua LRM, Rossato JI, Bonini J, Medina JH, Cammarota M (2006). Different molecular cascades in different sites of the brain control consolidation. Trends Neurosci.

[17] Gruart A, Muñoz MD, Delgado-García JM (2006). Involvement of the CA3–CA1 synapse in the acquisition of associative learning in behaving mice. J Neurosci.

[18] Whitlock JR, Heynen AJ, Shuler MG, Bear MF (2006). Learning induces long-term potentiation in the hippocampus. Science.

[19] Cammarota M, Bevilaqua LR, Viola H, Kerr DS, Reichmann B, Teixeira V, Bulla M, Izquierdo I, Medina JH (2002). Participation of CaMKII in neuronal plasticity and memory formation. Cell Mol Neurobiol.

[20] Levenson J, Weeber E, Selcher JC, Kategaya LS, Sweatt JD, Eskin A (2002). Long-term potentiation and contextual fear conditioning increase neuronal glutamate uptake. Nat Neurosci.

[21] Bevilaqua LR, Kerr DS, Medina JH, Izquierdo I, Cammarota M (2003). Inhibition of hippocampal Jun N-terminal kinase enhances short-term memory but blocks long-term memory formation and retrieval of an inhibitory avoidance task. Eur J Neurosci.

[22] Bevilaqua LR, Bonini JS, Rossato JI, Izquierdo LA, Cammarota M, Izquierdo I (2006). The entorhinal cortex plays a role in extinction. Neurobiol Learn Mem.

[23] Mercer TR, Dinger ME, Mattick JS (2009). Long non-coding RNAs: Insights into functions. Nat Rev Genet.

[24] Cabili MN, Trapnell C, Goff L, Koziol M, Tazon-vega B, Regev A, Rinn JL (2011). Integrative annotation of human large intergenic noncoding RNAs reveals global properties and specifi subclasses. Genes Dev.

